# The Case for an Early Biological Origin of DNA

**DOI:** 10.1007/s00239-014-9656-6

**Published:** 2014-11-26

**Authors:** Anthony M. Poole, Nobuyuki Horinouchi, Ryan J. Catchpole, Dayong Si, Makoto Hibi, Koichi Tanaka, Jun Ogawa

**Affiliations:** 1 Biomolecular Interaction Centre, School of Biological Sciences, University of Canterbury, Christchurch, 8140 New Zealand; 2 Allan Wilson Centre, University of Canterbury, Christchurch, 8140 New Zealand; 3 Division of Applied Life Sciences, Graduate School of Agriculture, Kyoto University, Kitashirakawa-Oiwakecho, Sakyo-ku, Kyoto, 606-8502 Japan; 4 Department of Molecular Bioscience and Bioengineering, University of Hawaii, Honolulu, HI 96822 USA

**Keywords:** Ribonucleotide reductase, Deoxyriboaldolase, Last Universal Common Ancestor, RNA world, DNA origins

## Abstract

All life generates deoxyribonucleotides, the building blocks of DNA, via ribonucleotide reductases (RNRs). The complexity of this reaction suggests it did not evolve until well after the advent of templated protein synthesis, which in turn suggests DNA evolved later than both RNA and templated protein synthesis. However, deoxyribonucleotides may have first been synthesised via an alternative, chemically simpler route—the reversal of the deoxyriboaldolase (DERA) step in deoxyribonucleotide salvage. In light of recent work demonstrating that this reaction can drive synthesis of deoxyribonucleosides, we consider what pressures early adoption of this pathway would have placed on cell metabolism. This in turn provides a rationale for the replacement of DERA-dependent DNA production by RNR-dependent production.

**Electronic supplementary material:**

The online version of this article (doi:10.1007/s00239-014-9656-6) contains supplementary material, which is available to authorized users.

## Introduction

The RNA world hypothesis posits that templated protein synthesis and DNA-based storage of genetic information evolved well after RNA was already established as a major catalyst and as the genetic material (Gilbert 1986; Yarus 2011). Chemical (“bottom-up”) efforts to understand the RNA world have centred on the path from prebiotic chemistry to a simple RNA world (Anastasi et al. 2007; Benner et al. 1989; Dworkin et al. 2003; Hud et al. 2013) as well as ascertaining whether the catalytic capabilities of RNA could support the complex chemical metabolism presumed to be central to an RNA-based stage of life (Yarus 2002). In contrast, biological research has focused on whether the evolutionary history of RNA molecules is consistent with their presence early in the history of life (Hoeppner et al. 2012; Jeffares et al. 1998; White 1976). Likewise, a key part of this “top-down” or biological approach to testing the RNA world hypothesis has been to understand the nature of the evolutionary transitions from RNA to protein and from RNA to DNA. That the ribosome contains universally conserved RNAs, integrally involved in decoding and peptidyl transfer (Nissen et al. 2000; Noller 2010; Petrov et al. 2014), provides compelling evidence in favour of the RNA world, as does the demonstration that RNase P, a universal enzyme (Collins et al. 2000) required for tRNA maturation, is a catalytic RNA (Guerrier-Takada et al. 1983).

**Fig. 1:**
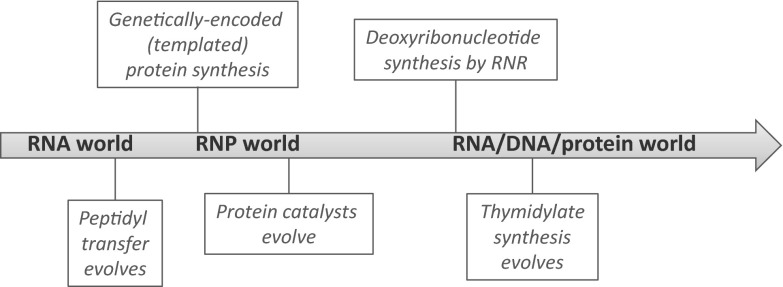
Overview of the evolutionary transitions from RNA to protein and DNA. One view of the transitions from the RNA world to the modern DNA + protein world holds that the first transition coincided with the origin of templated protein synthesis (Noller 2010). The transition to DNA as genetic material is thought to have occurred later, requiring the evolution of genetically encoded protein enzymes. This view is based on the catalytic complexity of ribonucleotide reduction, which places the modern reaction beyond the capability of catalytic RNA (Poole et al. 2000). The transition from RNA to DNA is thought to be a two-stage transition, with replacement of the fourth base being a later step, possibly driven by futile cycling of deamination repair (Poole et al. 2001), or a virus-driven coevolutionary arms race (Forterre 2002)

Equally, that all life synthesizes deoxyribonucleotides from ribonucleotide precursors via the ubiquitous (Lundin et al. 2009), structurally homologous (Larsson et al. 2004; Logan et al. 1999; Sintchak et al. 2002; Tauer and Benner 1997; Uhlin and Eklund 1994) catalytic subunit of ribonucleotide reductases (RNRs), suggests that deoxyribonucleotide synthesis was added onto an already established ribonucleotide synthesis pathway (Lazcano et al. 1988; Poole et al. 2001, 2002; Reichard 1993; Torrents et al. 2002). However, ribonucleotide reduction is chemically complex, requiring the generation and containment of free radicals (Hofer et al. 2012). This requirement for free-radical chemistry suggests that the ribonucleotide reduction reaction, as carried out by modern RNRs, was beyond the capacity of RNA enzymes because RNA is indiscriminately cleaved by free radicals (Poole et al. 2000). This in turn suggests the evolutionary transition from RNA to DNA was preceded by the emergence of genetically encoded protein catalysts (Freeland et al. 1999; Poole et al. 2000) (Fig. 1).

Whereas biologists primarily see evolution of ribonucleotide reduction as the main roadblock to evolution of DNA (Freeland et al. 1999; Poole et al. 2000), deoxyribonucleotide synthesis is considered from a chemical perspective to be relatively straightforward compared with evolving a ribosome (Benner et al. 1989; Burton and Lehman 2009). In contrast to the possibility of an early chemical origin for DNA, comparative genomics analyses can be interpreted as compatible with a late origin for DNA (Forterre 1999; Forterre 2002; Leipe et al. 1999), perhaps post-dating the divergence of modern cells from a common ancestor (often dubbed LUCA, for Last Universal Common Ancestor). That said, comparative data are also compatible with an earlier, pre-LUCA origin for DNA (Poole and Logan 2005). Consequently, top-down and bottom-up views have diverged significantly on this point. The overarching goal of origins research is to build a coherent picture spanning prebiotic chemistry through to early evolution. To this end, it is worth considering whether an early case for DNA can be made from the biological ‘top-down’ perspective, and how such a model might be tested. We first review the apparently opposing views that have emerged for both the top-down and bottom-up models, then we turn to whether they might converge on a consensus.

## A Top-Down Perspective on the Origin of DNA

While ribonucleotide reduction provides the sole mechanism for *de novo* synthesis of deoxyribonucleotides, the evolutionary history of RNRs, and even the DNA replication machinery, is complex. Consequently, it is difficult to place any complete DNA-associated processes in the LUCA (Forterre et al. 2004; Harris et al. 2003; Leipe et al. 1999). In stark contrast to ribosomal RNAs and many ribosomal proteins, which show a primarily vertical evolutionary history (Goldman et al. 2013; Harris et al. 2003; Woese and Fox 1977), the early evolutionary history of RNRs is peppered by interdomain horizontal transfer (Lundin et al. 2010), and there is too little sequence similarity to build sequence-based phylogenies spanning all three classes of RNRs (Lundin et al. 2010; Tauer and Benner 1997). The varying operational constraints of RNRs provide a clear explanation for this observation; RNRs have diversified into strictly anaerobic (class III), B12-dependent (class II, which operate irrespective of oxygen presence/absence) and two strictly aerobic forms (classes Ia & Ib) (Lundin et al. 2010). These classes are utilised in different environmental contexts (del Mar Cendra et al. 2012), and many microbes carry multiple classes of RNR (Lundin et al. 2009). Consequently, environment and horizontal transfer are key drivers of RNR inheritance patterns (Dwivedi et al. 2013; Lundin et al. 2010).

The other key enzymatic reaction central to the RNA to DNA transition is thymidylate synthesis (Fig. 1b). Thymidylate synthases, which convert dUMP to dTTP, were thought to have a single origin, but the discovery of a second class of thymidylate synthase (ThyX), unrelated to conventional thymidylate synthases (Myllykallio et al. 2002), shows that, while the reactions might be universal, the enzymes are not. The observation that the thymidylate synthase genes have distinct distributions, with the new class spread across a number of lineages, can best be understood in light of horizontal gene transfer (Myllykallio et al. 2002).

**Fig. 2:**
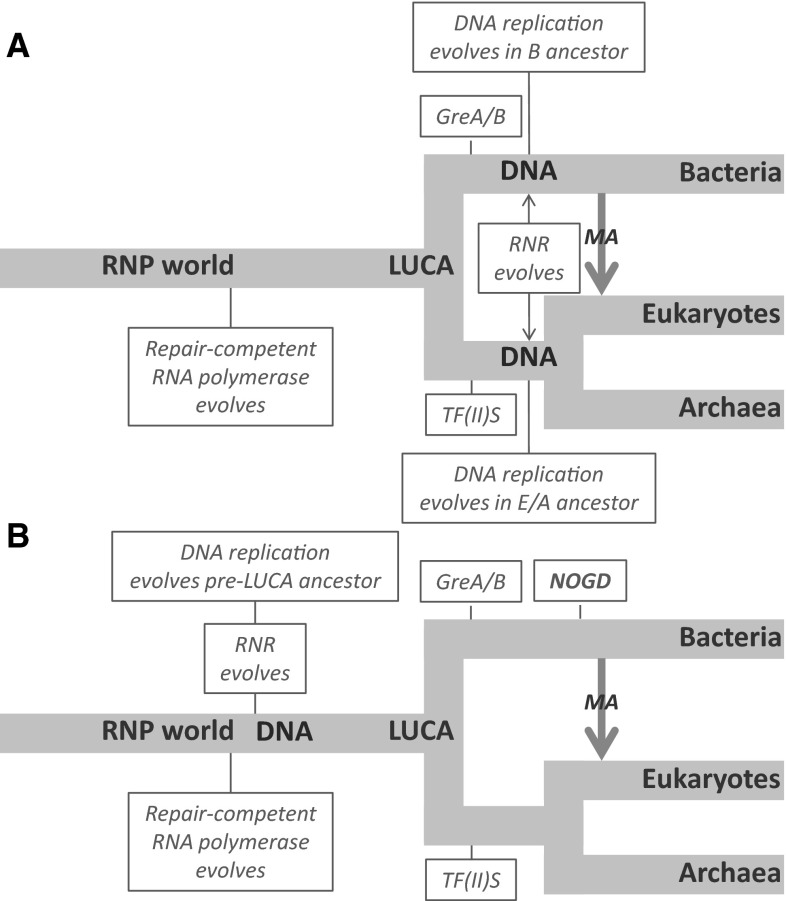
Two interpretations of the biological data on DNA origins. The observation that much of the core machinery for DNA replication differs between Bacteria on the one hand, and Archaea/Eukaryotes on the other (Forterre et al. 2004; Leipe et al. 1999) can be interpreted in two different ways (Poole and Logan 2005). **a** The case for an RNA-based LUCA can be made under a model where a repair-competent RNA polymerase is utilised in a replicative capacity, with lineage-independent origins for RNA proofreading fidelity factors (the GreA/B system in Bacteria, and the TF(II)S system in Archaea/Eukaryotes). Later, the replicative DNA apparatus emerges twice independently (Leipe et al. 1999). Under this model, the origin of ribonucleotide reduction is late but is obscured by subsequent horizontal gene transfer events (Lundin et al. 2010). **b** The case for a DNA-based LUCA can be made on the exact same data by invoking non-orthologous gene displacement (NOGD) (Leipe et al. 1999). For clarity, the bacterial lineage is depicted as being subject to NOGD, but this could equally have occurred in the Archaeal/Eukaryotic lineage. The key point of this model is that one of the two apparatuses is evolutionarily older than the other, with the displacement possibly occurring through acquisition of viral replication genes (Forterre 2013). This model fits with the observation that some features of the DNA replication machinery appear to be ubiquitous as do some parts of the DNA repair machinery. It also permits placement of ribonucleotide reduction before the divergence of the three domains (while still accepting modern transfer events obscure the deep origins of ribonucleotide reductase). It is also possible for RNA polymerase fidelity factors to be placed pre-LUCA under non-orthologous gene displacement (Poole and Logan 2005). *MA* mitochondrial ancestor. Note that there is ongoing debate regarding the exact relationship between eukaryotes and the Archaea (Poole and Gribaldo 2014). Each domain is shown as a distinct group for clarity, though under an archaeal origin for eukaryotes the interpretation of this figure is unchanged

A number of key parts of the DNA replication machinery exhibit similarly complex histories. Most notably, replicative DNA polymerases and primases in bacteria are unrelated to those in archaea and eukaryotes (Augustin et al. 2001; Bailey et al. 2006; Leipe et al. 1999), raising the possibility that modern processes for DNA synthesis and replication could have evolved after the primary bifurcation that gave rise to Archaea/Eukarya and Bacteria. In contrast, the conserved core of cellular RNA polymerases have a single evolutionary origin, and their evolutionary history appears broadly congruent with that of the ribosome (Cramer 2002; Tourasse and Gouy 1999; Werner and Grohmann 2011). A further series of observations that are consistent with a late origin for DNA are that modern RNA polymerases are capable of proofreading and repair and can replicate RNA templates (Lehmann et al. 2007; Poole and Logan 2005; Zenkin et al. 2006), notably in replication of plant viroids and Hepatitis Delta Virus (Fels et al. 2001; Lai 2005). This, coupled with an appreciation of the robustness of RNA to mutation (Kun et al. 2005), and the expected early origin of recombination (Lehman 2003; Reanney 1987), raises the possibility of large RNA genomes in early cellular lineages (Poole 2006; Poole and Logan 2005). In addition, accessory proteins which augment proofreading by RNA polymerase also evolved independently in Archaea/Eukarya and Bacteria after this primary diversification, suggesting that improvements to RNA fidelity were ongoing prior to the switch to use of DNA for genetic storage (Poole and Logan 2005). Together, these results point to a very late origin for DNA, post-dating the diversification of the primary lineages from the LUCA (Fig. 2a).

It is worth noting that, while the primary focus in thinking about the evolution of the genetic material is often on fidelity of replication, in light of the fact of RNA repair, an alternative model for the origin of DNA is at least as plausible, particularly in light of horizontal gene transfer of both replicative and synthetic enzymes. That model, proposed by Forterre, is that the enzymes required for synthesis and replication initially evolved in viruses. In this model, viral-host cell coevolution drove modification of viral genetic material, much as it does in modern viruses (Forterre 2002, 2006). These modifications served to provide viral genome protection from cellular defences (RNases in the first instance), and these host-virus interactions subsequently led to viral-to-cellular transfers, neutralising the viral advantage. Forterre’s model helps to account for the wide variety of enzymes that perform the same functions in deoxyribonucleotide synthesis and DNA replication (Forterre 2013), something that other models fail to comprehensively address.

## A Bottom-Up Perspective on the Origin of DNA

**Fig. 3:**
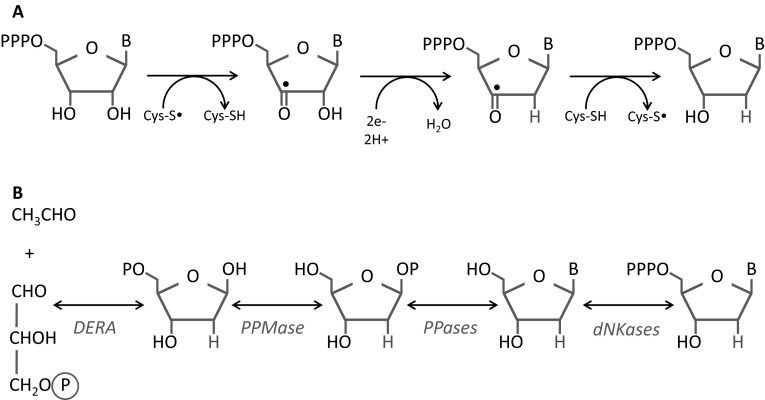
The chemistry of ribonucleotide reduction and deoxyribonucleotide salvage. **a** Generalised pathway for ribonucleotide reduction, showing steps common to all ribonucleotide reductase classes. Production of a stable cysteinyl radical in the active site of ribonucleotide reductase occurs in all variants of ribonucleotide reduction, though the initial formation of the radical differs across classes. The radical is transferred to the ribonucleotide substrate (depicted here as an NTP, though this can be either NDP or NTP, depending on the organism), driving protonation of the 2′ hydroxyl group, with the release of a water molecule. This step involves a separate reductant, and the nature of the reductant differs between ribonucleotide reductase classes. Finally, the cysteinyl radical is regenerated before being returned to the original site of radical generation (cofactor or a different protein residue). See (Hofer et al. 2012) for a recent review of ribonucleotide reduction. **b** Deoxyribonucleosides can be produced from glyceraldehyde-3-phosphate and acetaldehyde via the reverse of the deoxyriboaldolase (DERA) reaction, forming 2-deoxyribose-5-phosphate. The subsequent action of phosphopentomutase (PPMase), phosphorylases (PPases) and deoxynucleotide kinases (dNKases) generates dNTPs. Modern enzymes can drive the synthesis of deoxyribonucleosides but have thus far only been shown to do so in vitro (Horinouchi et al. 2006c)

The reverse deoxyriboaldolase (DERA) reaction (Fig. 3) (Racker 1951, 1952) has been noted by prebiotic chemists (Benner et al. 1989; Burton and Lehman 2009) as a chemically far simpler—and therefore possibly much earlier—route to deoxyribonucleotide synthesis. DERA is involved in modern salvage, through which deoxyribonucleosides are catabolised to acetaldehyde and glyceraldehyde-3-phosphate, where they can be incorporated into central metabolism. However, prior to the discovery of ribonucleotide reduction (Reichard and Rutberg 1960), the reverse DERA pathway was considered the most likely cellular route for deoxyribonucleotide synthesis (Racker 1951, 1952). That said, owing to low intracellular levels of acetaldehyde, the reaction is only known to proceed in the degradative direction in vivo, and synthesis is only observed in cell extracts (Horinouchi et al. 2006c; Racker 1952).

As elegantly laid out by Burton and Lehman (Burton and Lehman 2009), all the steps in the reaction outlined in Fig. 3b could plausibly be performed by ribozyme chemistry, suggesting a bottom-up approach to this question could bear experimental fruit. In contrast, to be within reach of ribozyme chemistry, ribonucleotide reduction would need to proceed via an alternative chemical route (Burton and Lehman 2009) as the chemistry employed by modern RNRs (Fig. 3a) remains, on current knowledge, beyond the reach of ribozymes (Poole et al. 2000). Thus, if DNA usage did evolve early, the ribozyme-based DERA pathway currently provides the most plausible chemical route.

## Building a Case for the Early Origin of DNA via the DERA Pathway

An important goal for origins research is to find connections between emerging biological and chemical insights. At first glance, the data underlying the top-down model, where DNA evolved late (Fig. 2a), appear incompatible with an early origin for DNA via reverse DERA. However, as with all biological reconstructions of the deep past, these events are clouded by uncertainty, and more than one interpretation is possible. Indeed, by invoking non-orthologous gene displacement events (Leipe et al. 1999), the biological data can be shown to be equally compatible with an earlier origin for DNA, and it is in principle possible to place DNA in the LUCA (Forterre 2013; Poole 2011; Poole and Logan 2005) (Fig. 2b).

That comparative analysis as a general approach is limited in its capacity to produce a definitive picture is well established. Reconstructions of the genomic content of the LUCA show that, beyond the ribosome, a smattering of ribosome-associated processes and the conserved core of multisubunit RNA polymerases, it is difficult to consistently trace many more features to the LUCA (Goldman et al. 2013; Harris et al. 2003; Hoeppner et al. 2012; Koonin 2003). These processes are clearly insufficient to run a cell, and there is widespread acceptance that horizontal gene transfer and non-orthologous gene displacement must have played a role in the shaping of modern biology (Koonin 2003; Nelson-Sathi et al. 2014; Poole 2009; Vetsigian et al. 2006; Woese 2002). Loss of information is an equally large problem for reconstructing past states, both in terms of gene losses, which risk being interpreted as evidence for late emergence (Becerra et al. 1997; Glansdorff et al. 2008), and exponential loss of signal in sequence data, which limits phylogenetic reconstruction based on sequence data (Penny and Zhong 2014) (though structural information can help here (Daly et al. 2013; Lundin et al. 2012).

In light of these processes, the LUCA could well have carried a full set of DNA replication machinery, and in this regard it is noteworthy that key parts of the DNA replication machinery do appear universal. The clamp-loader and clamp, which give DNA polymerases their processivity, are universally conserved (Kelch et al. 2012; Leipe et al. 1999), as is RNase HII, which facilitates removal of RNA primers used during replication (Brindefalk et al. 2013; Tadokoro and Kanaya 2009). Clamp-loader, clamp and RNase H genes are also found in viruses, consistent with the viral-cellular coevolution model for DNA origins (Forterre 2013). The recent discovery that the universally conserved replicative helicase, UvrD, is intimately involved in recruiting the DNA repair machinery (some of which has also been argued to trace to the LUCA (Eisen and Hanawalt 1999)) at the site of active transcription (Epshtein et al. 2014), is also compatible with a DNA-based LUCA. Moreover, that RNRs, thymidylate synthases, and even DNA polymerases, are subject to ongoing horizontal gene transfer and non-orthologous gene displacement, including between viruses and their hosts, suggests that the machinery involved in deoxyribonucleotide synthesis and DNA replication cannot be interpreted exclusively in terms of late gains.

Finally, support for an early origin of DNA has also emerged via modelling. In simulation studies, it was shown that a DNA-like capacity is advantageous early because it eliminates the trade-off between the information storage and catalytic functions of RNA, and in fact makes the modelled system more robust to invasion by parasites (Takeuchi et al. 2011).

If the interpretation in Fig. 2b is accepted, then the mutual incompatibility of a very early origin for DNA and comparative analyses vanishes. Such an interpretation removes the difficulty of placing DNA in the LUCA, though it does not enable us to directly reconstruct the replicative machinery of this early stage.

**Fig. 4:**
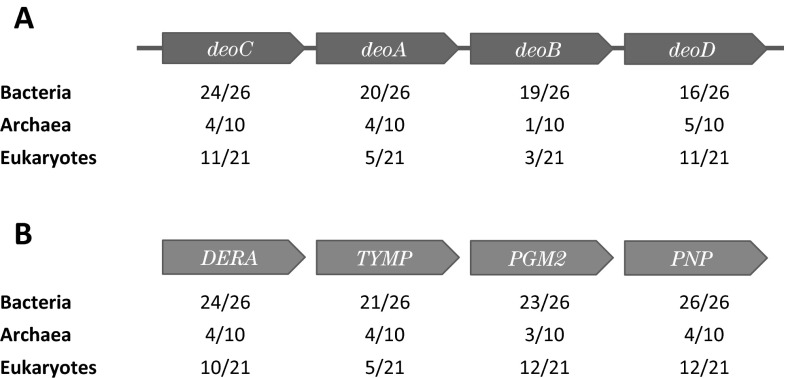
The deoxyriboaldolase pathway is present in all three domains. **a** The proteins encoded by the *E. coli deo* operon were used as query sequences for a pHMMER search (Finn et al. 2011) against the nr protein database. Red arrows indicate ORFs and their position in the *E. coli deo* operon. The number of phyla in each domain with significant hits (*E*-value <0.01) were counted (see Tables S1–3 for phylum-level results). **b** Genes from the *H. sapiens* genome with identical function to those of the *E. coli deo* operon are shown in blue. The proteins encoded by these genes were used as query sequences for a pHMMER search against the nr protein database. The number of phyla in each domain with significant hits (*E*-value <0.01) were counted (see Tables S1–3 for phylum-level results)

Plausibility is important, but biologists are equally concerned with historical signal. Significantly, RNRs are ubiquitous (bar a handful of bacteria, which lost RNRs during adaptation to an obligate intracellular lifestyle and now derive deoxyribonucleotides from their host) (Lundin et al. 2010, 2009). Thus, if the reverse DERA reaction ever was used for deoxyribonucleotide synthesis, it has long ceased to perform this function. That said, salvage via DERA is widespread, and the key genes required for operation of this pathway can be readily identified in all three domains (Fig. 4).

## Towards Testing Deep Evolution in a Cellular, Experimental Context

If DNA synthesis and replication are placed in the LUCA, could we take the next step and consider the reverse deoxyriboaldolase reaction as a possible early route to DNA? This has yet to be investigated. Certainly, the distribution of the enzymes is compatible with an ancient origin (Fig. 4), but more crucially, biology is now developing tools (Elena and Lenski 2003; Gibson et al. 2010; Lutz and Patrick 2004) to enable hypothesis testing on a level that matches the incredible success of SELEX experiments in expanding the chemistry of the RNA world (Breaker and Joyce 2014; Yarus 1999).

There are several important ways that biology may help connect to chemical research into DNA origins. We lay these out as a series of general questions that could be applied to the empirical study of any pathway:

1. Can the product be produced by existing enzymes?
2. Can the product be produced in vivo by existing enzymes?
3. Can the reaction replace the incumbent pathway?

For DNA origins, the answer to the first question is straightforward: the reverse DERA reaction was known to operate at the time that deoxyriboaldolase was first characterised (Racker 1951, 1952). That said, there is an important parallel between SELEX experiments to test the feasibility of the individual steps in this pathway in a hypothetical RNA world (Burton and Lehman 2009) and microbial process engineering. Here, screening has yielded novel natural versions of these enzymes that, together, can improve the synthetic yield of the desired product (Horinouchi et al. 2006a, b, c, 2003, 2012; Ogawa et al. 2003). Thus, pathway optimisation may be rapidly achieved through creation of a patchwork of enzymes from different species.

SELEX and directed evolution of proteins test one enzyme or reaction at a time. The patchwork approach thus helps test more complex processes in a cell-free environment, such as deoxyribonucleoside synthesis (Horinouchi et al. 2006b), and even simplified 19-amino acid genetic codes (Kawahara-Kobayashi et al. 2012). These examples address Question 1 above in that they show that a process can be carried out by the components of biological systems.

In the case of undertaking replacement of one reaction by another, the solutions to questions 2 and 3 above are likely to be linked. Question 2 is vitally important because it asks whether a reaction will work in the complex context of a cell. In the case of deoxyribonucleotide synthesis, this permits us to address the original critique of reverse deoxyriboaldolation as a route to deoxyribonucleosides: that there would be insufficient starting substrate to ever drive the reaction in the direction of synthesis. A complication with an essential process such as deoxyribonucleotide synthesis is that knocking out the genes for the existing pathway (Question 3) can only be successful in the context of success in production by an alternative route (Question 2). Certainly, under normal circumstances, it is not possible to knock out all RNRs in *E. coli* simultaneously because this pathway is essential, even under conditions where a bespoke deoxyriboaldolase operon is overexpressed (DS, NH, RJC personal observations). Consequently, the challenge in testing the biological viability of the reverse DERA pathway is finding conditions that should favour synthesis.

Analysis of the DERA pathway suggests the key problem is acetaldehyde. Acetaldehyde is highly reactive and may not be available in vivo in sufficient amounts for synthesis. Moreover, the levels of acetaldehyde used in microbial process engineering for deoxyribonucleoside synthesis are toxic to *E. coli* (Horinouchi et al. 2006a; Ogawa et al. 2003). Additionally, acetaldehyde is important for NAD+ regeneration. *E. coli* carries multiple alcohol dehydrogenase genes, which reduce acetaldehyde to ethanol, oxidising NADH in the process. Thus, if acetaldehyde is diverted from NAD+ production, this may upset NAD+/NADH pools. All of these hurdles would need to be negotiated in testing the possibility of the DERA pathway supporting deoxyribonucleotide synthesis in a cellular system.

## Rapid Takeover of Deoxyribonucleotide Synthesis by Ribonucleotide Reduction

There are good reasons to expect that, if early cells did utilise deoxyriboaldolase for deoxyribonucleotide synthesis, this pathway was unlikely to persist as a primary synthetic route following the advent of ribonucleotide reduction. We will first consider the pros of modern ribonucleotide reduction, before comparing this to the DERA pathway.

Modern deoxyribonucleotide pools are one to two orders of magnitude lower than nucleotide pools (Nick McElhinny et al. 2010), so diverting a small fraction of the latter to deoxyribonucleotide synthesis following advent of ribonucleotide reduction likely caused minimal metabolic disruption to the cell. Ribonucleotide reduction is an irreversible reaction, and RNRs exhibit sophisticated allosteric regulation (as does pyruvate formate lyase, which is homologous to RNRs, and appears evolutionarily closest to class III RNRs (Leppanen et al. 1999; Logan et al. 1999)). Hence, modern deoxyribonucleotide synthesis can be tightly controlled, thereby avoiding unbalanced or elevated dNTP levels, which would lead to increased mutation rates (Hofer et al. 2012).

In contrast to ribonucleotide reduction, the DERA reaction is reversible. Therefore, there is potential for futile cycling. If deoxyribonucleotides were initially synthesised via the DERA pathway, deoxyribonucleotide pools may have fluctuated based on whether substrates (acetaldehyde and glyceraldehyde-3-phosphate) or product (deoxyribonucleosides) were in abundance (Horinouchi et al. 2006c; Ogawa et al. 2003). Given the toxicity of acetaldehyde—which, in modern cells causes DNA damage, formation of protein adducts, and free radical production (Dellarco 1988)—2-deoxyribose-5-phosphate production may have initially provided a means of eliminating this toxic molecule. However, without modification of 2-deoxyribose-5-phosphate, the resulting low levels of acetaldehyde could drive breakdown of deoxyribonucleosides. Downstream reactions (Fig. 3) could have favoured deoxyribonucleotide synthesis, and reduced futile cycling, but the entire reaction is still reversible, which would have resulted in poor control of deoxyribonucleotide pools, a phenomenon that is known in modern systems to lead directly to mutational pressures (Yao et al. 2013). Finally, if the DERA pathway operated in the direction of synthesis, there may have been competition between DERA-driven deoxyribonucleotide synthesis on the one hand, and use of acetaldehyde as an electron acceptor for NAD+ regeneration (producing ethanol) on the other.

In contrast, ribonucleotide reduction would have offered efficient, irreversible deoxyribonucleotide formation, which would have been critical to reducing futile cycling, and would have provided better control of deoxyribonucleotide synthesis. Utilising a small fraction of the ribonucleotide pool would have had minimal disruption on existing roles for this substrate, and permitted more effective NAD+ regeneration through fermentation. Thus, if the DERA pathway did evolve much earlier than ribonucleotide reduction, comparison of the two pathways suggests DERA would be completely superseded as a synthetic pathway following the advent of ribonucleotide reduction.

## Concluding Remarks

There has been enormous progress in understanding the evolutionary origins of DNA. However, different views have been reached based on assessment of the evolutionary history of the cellular apparatus for deoxyribonucleotide synthesis and DNA replication (Forterre 2002; Leipe et al. 1999; Poole and Logan 2005) or from chemical considerations (Burton and Lehman 2009). One interpretation of the biological data is that DNA may have evolved after Bacteria diverged from Archaea and Eukaryotes, such that the LUCA possessed an RNA genome. However, the data could also be consistent with a DNA-based LUCA, but with DNA evolving after the advent of complex protein-based catalysts. In contrast, if chemical routes to deoxyribonucleotides are not limited to ribonucleotide reduction, then DNA could have emerged in an RNA world, before templated protein synthesis. While these views appear diametrically opposed, it is not clear that these they are actually incompatible (Poole 2011). Indeed, we see no reason that an early RNA world origin of deoxyribonucleotide synthesis cannot be compatible with a much later origin of the modern enzymes for deoxyribonucleotide synthesis. A promising avenue to address this is to establish whether it is possible to create the conditions wherein a cell can synthesize deoxyribonucleotides via acetaldehyde and glyceraldehyde-3-phosphate instead of via ribonucleotide reduction. It is clear that modern DERA pathway enzymes can indeed drive deoxyribonucleoside synthesis (Horinouchi et al. 2006a), and this shows great promise for industrial-scale production of deoxyribonucleosides (Horinouchi et al. 2012). From an evolutionary perspective, assessment of the capacity for this pathway to replace ribonucleotide reduction in vivo is the crucial next step.

## Electronic supplementary material

Below is the link to the electronic supplementary material.

Supplementary material 1 (DOCX 26 kb)
