# Supplementary material for: The Case for an Early Biological Origin of DNA
Source: J Mol Evol. 2014 Nov 26;79(5):204–12. doi: 10.1007/s00239-014-9656-6 (PMC4247479; doi:10.1007/s00239-014-9656-6)
Supplement: Supplementary file 1 — Supplementary material 1 (DOCX 26 kb) [file 239_2014_9656_MOESM1_ESM.docx]

Supplementary material for: **The case for an early biological origin for DNA.**

**Table S1:** Archaeal phylum-level summary of phmmer search results (E value <0.01) using proteins coded by the *E. coli deo* operon and their *H. sapiens* equivalents (in parentheses), against the nr protein database.

|  | deoA  (TYMP) | | deoB  (PGM2*) | | deoC  (DERA) | | deoD  (PNP) | |
| --- | --- | --- | --- | --- | --- | --- | --- | --- |
| Aenigmarchaeota |  |  |  |  |  |  |  |  |
| Crenarchaeota | 10 | (13) | 1 | (56) | 33 | (28) | 92 | (48) |
| Diapherotrites |  |  |  |  |  |  |  |  |
| Euryarchaeota | 112 | (190) | 0 | (320) | 146 | (122) | 231 | (190) |
| Geoarchaeota |  |  |  |  |  |  |  |  |
| Korarchaeota |  |  |  |  |  |  | 1 | (1) |
| Nanoarchaeota | 1 | (1) |  |  |  |  | 1 | (0) |
| Nanohaloarchaeota |  |  |  |  |  |  |  |  |
| Parvarchaeota |  |  |  |  | 2 | (1) |  |  |
| Thaumarchaeota | 2 | (4) | 0 | (19) | 1 | (1) | 3 | (15) |

*The equivalent of *deoB* in *H. sapiens* is *PGM2*, a phosphoglucomutase with phosphopentamutase activity (Maliekal et al. 2007), which thus returns greater numbers of hits (to phosphoglucomutase homologs).

**Table S2:** Bacterial phylum-level summary of phmmer search results (E value <0.01) using proteins coded by the *E. coli deo* operon and their *H. sapiens* equivalents (in parentheses), against the nr protein database.

|  | deoA  (TYMP) | | deoB  (PGM2*) | | deoC  (DERA) | | deoD  (PNP) | |
| --- | --- | --- | --- | --- | --- | --- | --- | --- |
| Actinobacteria | 839 | (954) | 58 | (1553) | 954 | (928) | 336 | (1049) |
| Aquificae | 2 | (19) | 0 | (23) | 14 | (14) | 1 | (26) |
| Armatimonadetes | 3 | (4) | 2 | (2) | 2 | (2) | 0 | (3) |
| Bacteroidetes/Chlorobi | 31 | (33) | 6 | (1044) | 586 | (561) | 564 | (624) |
| Caldiserica | 1 | (0) | 1 | (0) | 1 | (1) | 1 | (2) |
| Chlamydiae/Verrucomicrobia | 14 | (14) | 2 | (119) | 18 | (18) | 1 | (14) |
| Chloroflexi | 10 | (15) | 2 | (28) | 13 | (13) | 7 | (37) |
| Chrysiogenetes |  |  |  |  |  |  | 0 | (2) |
| Cyanobacteria | 1 | (6) | 0 | (312) | 137 | (121) | 37 | (62) |
| Deferribacteres |  |  | 0 | (7) | 1 | (1) |  | (6) |
| Deinococcus-Thermus | 36 | (54) | 39 | (77) | 41 | (41) | 56 | (25) |
| Dictyoglomi | 2 | (4) | 2 | (2) | 2 | (2) | 0 | (2) |
| Elusimicrobia | 0 | (1) | 1 | (4) | 1 | (1) | 0 | (3) |
| Fibrobacteres/Acidobacteria | 13 | (20) | 2 | (41) | 19 | (19) | 0 | (32) |
| Firmicutes | 2245 | (2894) | 2427 | (4607) | 2810 | (2648) | 3040 | (1674) |
| Fusobacteria | 18 | (18) | 26 | (158) | 35 | (35) | 153 | (8) |
| Gemmatimonadetes | 3 | (5) | 4 | (0) | 3 | (3) | 0 | (5) |
| Nitrospinae |  |  | 0 | (5) |  |  | 0 | (2) |
| Nitrospirae |  |  | 1 | (19) | 1 | (1) | 0 | (9) |
| Planctomycetes | 14 | (22) | 0 | (29) | 19 | (18) | 1 | (39) |
| Proteobacteria | 1881 | (3874) | 1914 | (3812) | 1915 | (1900) | 2966 | (1796) |
| Spirochaetes | 55 | (61) | 24 | (391) | 65 | (63) | 121 | (45) |
| Synergistetes | 10 | (12) | 2 | (12) | 11 | (13) | 4 | (15) |
| Tenericutes | 141 | (140) | 64 | (112) | 125 | (125) | 164 | (4) |
| Thermodesulfobacteria | 0 | (2) | 0 | (4) | 2 | (2) | 0 | (4) |
| Thermotogae | 21 | (21) | 15 | (32) | 25 | (25) | 16 | (41) |

*The equivalent of *deoB* in *H. sapiens* is *PGM2*, a phosphoglucomutase with phosphopentamutase activity (Maliekal et al. 2007), which thus returns greater numbers of hits (to phosphoglucomutase homologs).

**Table S3:** Eukaryotic phylum-level summary of phmmer search results (E value <0.01) using proteins coded by the *E. coli deo* operon and their *H. sapiens* equivalents (in parentheses), against the nr protein database.

|  | deoA  (TYMP) | | deoB  (PGM2*) | | deoC  (DERA) | | deoD  (PNP) | |
| --- | --- | --- | --- | --- | --- | --- | --- | --- |
| Alveolata | 2 | (2) | 0 | (56) | 41 | (39) | 72 | (8) |
| Amoebozoa |  |  | 0 | (18) | 7 | (7) | 23 | (1) |
| Apusozoa |  |  |  |  |  |  |  |  |
| Breviatea |  |  |  |  |  |  |  |  |
| Centroheliozoa |  |  |  |  |  |  |  |  |
| Cryptophyta | 1 | (1) | 0 | (1) | 1 | (1) | 0 | (1) |
| Euglenozoa |  |  | 0 | (16) | 20 | (20) | 10 | (14) |
| Fornicata |  |  | 0 | (10) | 5 | (6) | 7 | (6) |
| Glaucocystophyceae |  |  |  |  |  |  |  |  |
| Haptophyceae |  |  |  |  |  |  | 1 | (0) |
| Heterolobosea |  |  | 0 | (1) | 1 | (1) | 1 | (1) |
| Jakobida |  |  |  |  |  |  |  |  |
| Katablepharidophyta |  |  |  |  |  |  |  |  |
| Malawimonadidae |  |  |  |  |  |  |  |  |
| Opisthokonta | 143 | (154) | 7 | (645) | 345 | (387) | 51 | (941) |
| Metazoa | 139 | (151) | 7 | (432) | 227 | (232) | 6 | (745) |
| Oxymonadida |  |  |  |  |  |  |  |  |
| Parabasalia |  |  | 0 | (5) | 2 | (2) | 8 | (1) |
| Rhizaria |  |  | 0 | (2) | 2 | (1) | 0 | (4) |
| Rhodophyta |  |  | 0 | (3) |  |  | 0 | (1) |
| Stramenopiles | 2 | (4) | 0 | (24) | 22 | (22) | 6 | (21) |
| Viridiplantae | 1 | (1) | 2 | (8) |  |  | 3 | (2) |

*The equivalent of *deoB* in *H. sapiens* is *PGM2*, a phosphoglucomutase with phosphopentamutase activity (Maliekal et al. 2007), which thus returns greater numbers of hits (to phosphoglucomutase homologs).

**Reference:**

Maliekal P, Sokolova T, Vertommen D, Veiga-da-Cunha M, Van Schaftingen E (2007) Molecular identification of mammalian phosphopentomutase and glucose-1,6-bisphosphate synthase, two members of the alpha-D-phosphohexomutase family. J Biol Chem 282:31844
